# Heavy metal content in the green fodder of field pea/oat mixtures destined for cattle feed

**DOI:** 10.1007/s10661-019-7874-5

**Published:** 2019-10-26

**Authors:** Anna Płaza, Barbara Gąsiorowska, Emilia Rzążewska

**Affiliations:** Siedlce University of Natural Sciences and Humanities, Faculty of Agrobioengineering and Animal Husbandry, Agriculture and Gardening Institutes, Research Team of Agrotechnology, Prusa 14, 08-110 Siedlce, Poland

**Keywords:** *Pisum sativum*, *Avena sativa*, Mixture, Harvest date, Heavy metals

## Abstract

The objective of the study was to establish the effect of component share in mixtures and harvest date on concentrations of selected heavy metals in the green fodder of field pea, oat, and their mixtures. The research hypothesis assumed that the cultivation of peas and oats in pure sowing, and in mixtures will also allow to choose combinations from which the forage will have the lowest content of heavy metals. Field research was conducted at the Zawady Experimental Farm (52° 03′ 39″ N, 22° 33′ 80″ E) which belongs to Siedlce University of Natural Sciences and Humanities. Two factors were tested in the study: I—component share in the mixture: field pea—pure stand 100%, oat—pure stand 100%, field pea 75% + oat 25%, field pea 50% + oat 50%, field pea 25% + oat 75%; II—harvest date: field pea flowering stage, field pea flat pod stage. Plant material was sampled to determine the following elements: Cu, Zn, Cd, Pb, Cr, and Ni. The results of the study demonstrated that field pea grown in pure stand had the highest copper and zinc contents, and the lowest chromium and nickel contents. Field pea mixed with oat significantly reduced heavy metal content in green fodder. Cadmium and lead contents in the green fodder of field pea/oat mixtures were too low to be determined by means of the spectrometer Perkin Elmer Optima 8300. Regular checks of heavy metal contents are recommended in spite of their low amounts in the green fodder of field pea/oat mixtures.

## Introduction

Green fodder produced from legume/cereal mixtures is a valuable source of dairy cattle feed, providing it has a beneficial chemical composition, including a low concentration of heavy metals (Ociepa-Kubicka and Ociepa 2012; Chandra Sekhar et al. 2002). Thus, it is necessary to check its safety in terms of impurities present in feeds, including above-ground parts. Monitoring of toxic and potentially toxic elements is one of the most important aspects of maintaining feed quality. Transfer of heavy metals from soil to animal body occurs through plants which are the most important link in the food chain. Heavy metals contaminating the soil hamper the development of soil microorganisms. Negative correlations between the biomass of microorganisms and Pb, Zn, and Cu contents in soils contaminated with metals have been reported by Ociepa-Kubicka and Ociepa 2012 as well as Sharma et al. 2004. Heavy metal uptake by plants occurs through the rooting system and leaf blades. Heavy metals display various harmful effects. Lead, cadmium, chromium, and nickel are perceived as toxic metals but zinc and copper are microelements which are harmful when present in excess whereas their low amounts are necessary for the appropriate functioning of the body (Ali and Al-Qahtani 2012; Abdulmojeed and Abdulrahman 2011; Sharma et al. 2004). The following plants have the greatest capacity to accumulate heavy metals: lettuce, cabbage, beetroot, carrot, spinach, and potato, whereas the uptake by tomato, cucumber, leguminous, and cucurbitaceous vegetables as well as fruit is much lower. Cereal grain, and as a result green fodder, can be contaminated as well (Ali and Al-Qahtani 2012; Sobucola et al. 2010). Too high levels of metals in plants destined for animal feed may lead to animal-derived products being contaminated by the metals. Legumes are of particular importance as, when mixed with cereals, they reduce the uptake of heavy metals from the soil and limit their concentration in the soil (Dabney et al. 2001; Snapp et al. 2005). There is a perceptible lack of studies on the aforementioned subject. Thus, the need arises to undertake research in this area to monitor heavy metals in animal feed. The objective of the study reported here was to determine the effect of component share in the mixture and harvest date on the concentration of selected heavy metals in the green fodder of field pea, oat, and their mixtures.

## Materials and methods

Field research was conducted in the years 2010 to 2012 at the Zawady Experimental Farm (52° 03′ 39″ N, 22° 33′ 80″ E) which belongs to Siedlce University of Natural Sciences and Humanities. The experimental soil was Albic Luvisol (Arenic). The concentration of elements in the soil was as follows: P 5.21 mg 100 g^−1^, K 11.3 mg 100 g^−1^, Cu 1.3 mg 100 g^−1^, Zn 7.8 mg 100 g^−1^, Pb < 15.1 mg 100 g^−1^, Cd 0.10 mg 100 g^−1^, Cr< 12.6 mg 100 g^−1^, Ni < 12.6 mg 100 g^−1^. Soil reaction was neutral and humus content amounted to 1.39%. The experimental design was a split-block arrangement with three replicates. The area of plots under winter wheat was 30 m^2^ (5 m × 6 m), and the harvested area was 20 m^2^ (4 m × 5 m). Two factors were tested in the study: I—component share in the mixture: field pea—pure stand 100%, oat—pure stand 100%, field pea 75% + oat 25%, field pea 50% + oat 50%, field pea 25% + oat 75%; II—harvest date: field pea flowering stage, field pea flat green pod stage. The species proportion in a mixture was established in relation to the number of seeds planted in pure stands, that is 560 grains of oat and 120 grains of field pea m^2^. The following sowing rates were used: field pea 170 kg ּha^−1^, oat 180 kg ּha^−1^, field pea 128 kgּ ha^−1^ + oat 45 kg ּha^−1^, field pea 85 kg ּha^−1^ + oat 90 kg ּha^−1^, field pea 43 kg ּha^−1^ + oat 135 kg ּha^−1^.

In all the study years, the mixtures were preceded by winter triticale. Phosphorus and potassium fertilisers were applied in autumn and their rates, 35.2 kg ּha^−1^ P in the form of granular triple superphosphate and 99.6 kg ּha^−1^ K in the form of 60% potassium salt, depended on soil chemical composition. In spring, nitrogen fertiliser, at the rate of 30 kg ּha^−1^ N in the form of ammonium nitrate, was applied preplant to all the plots, excluding the units assigned to field pea grown in pure stand. At the stage of stem elongation, additional N was applied (50 kg ּha^−1^ for oat and 30 kg ּha^−1^ for field pea/oat mixtures). Field pea and oat seeds were planted in early April as described for the first experimental factor. Plants were harvested in late June and early July. During harvest of mixtures, fresh matter samples were taken from each plot to determine microelements. Cu, Zn, Cd, Pb, Cr, and Ni contents were determined by means of inductively coupled plasma optical emission spectrometry (ICP-OES) using the spectrometer Perkin Elmer Optima 8300.

Each characteristic tested was subjected to analysis of variance suitable for the split-block design. When significant sources of variation were confirmed, their means were separated using Tukey test. Calculations were performed in MS Excel 12.0.

The years of the study were characterised by significantly changeable weather conditions (Table 1). In 2010, mean air temperatures during the growing period fluctuated around the mean long-term temperatures. The precipitation totals, except for April, were higher than the mean long-term total precipitation. This year should be regarded as favourable for the cultivation of mixtures of field peas with oat. In 2011, the mean monthly air temperatures slightly differed from the mean long-term temperatures. However, the rainfall totals were lower than the mean long-term totals, except for July, where the recorded precipitation was 120.2 mm. In 2012, the average air temperature and total precipitation fluctuated around the long-term average. The lowest content of heavy metals was recorded in the green mass of mixtures of pea and oats in 2010, with the highest rainfall in June and July, and the highest content of heavy metals in 2012 with the lowest rainfall in July and throughout the growing season.

**Table 1:** Pluvio-thermal conditions in the growing season of pea and oat mixtures in 2010–2012 according to the Meteorological Station in RSD Zawady

Year	Month				Mean
	April	May	June	July	
Temperature °C					
2010	8.9	14.0	17.4	21.6	15.5
2011	10.1	13.4	18.1	18.3	15.0
2012	8.9	14.6	16.3	20.7	15.1
Long-term mean 1990–2005	8.2	14.2	17.6	19.7	14.9
Precipitation, mm					
2010	10.7	93.2	62.6	77.0	243.5
2011	31.0	36.1	39.1	120.2	226.4
2012	29.9	53.4	76.2	43.0	202.5
Long-term mean 1990–2005	37.4	47.1	48.1	65.5	198.1

## Results and discussion

Copper content in the green matter of field pea/oat mixtures was significantly affected by the experimental factors and their interaction (Table 2). The highest copper content (4.660 mg ּkg^−1^ d.m.) was recorded in field pea grown in pure stand, it being the lowest in oat (3.811 mg ּkg^−1^ d.m.). Also, Woźniak and Soroka (2016), Jarecki and Bobrecka-Jamro (2015), and Ali and Al-Qahtani (2012) observed a lower copper content in cereal grain rather than soy bean seed. In the experiment reported here, copper content in field pea, although higher than in oat, did not exceed the WHO/FAO standards, such feed being safe for animals. It should be emphasised that more copper was accumulated by legumes, although its amounts were low and did not pose a threat to human and animal health (Adefarati et al. 2017; Chandra Sekhar et al. 2002; Zarcinas et al. 2004). Plants take up small amounts of copper which, transferred to animals eating the plants, is necessary for the body because it participates in oxidation-reduction processes where it is a component of the coenzyme, and regulates metabolism, iron transportation, and collagen metabolism (Cabrera et al. 2003). In the present study, copper content in field pea/oat mixtures was significantly lower than in field pea grown in pure stand. The lowest Cu concentration was recorded in the mixture composed of the following respective shares of field pea and oat: 50 + 50% and 25 + 75%. A similar relationship was observed by Trąba and Wolański (2003) who reported that copper content in the dry matter of a grass/legume mixture was about twice as low as in legumes grown in pure stand. In the study reported here, harvest date had a significant influence on copper content in the green fodder of field pea/oat mixtures. A higher concentration of copper was recorded in field pea/oat mixtures harvested at the stage of field pea flowering compared with the stage of flat green pod. Research by Ladipo and Doherty (2011) as well as Abdulmojeed and Abdulrahman (2011) demonstrated that copper uptake by leafy vegetables is affected by such factors as climate, precipitation, concentration of heavy metals in soil, soil type, and maturity of plants at harvest. Fytianos et al. (2001), Demirezen and Aksoy (2006), and Muchuweti et al. (2006) have claimed that plants harvested at earlier development stages contain more copper. In the present study, an interaction between the experimental factors was confirmed indicating that field pea grown in pure stand and harvested at flowering contained the highest amount of copper, it being the lowest in pure stand oat harvested at the stage of flat green pod. All the test field pea/oat mixtures harvested at this stage had a lower copper content compared with field pea cultivated in pure stand and harvested at the flat green pot stage.

**Table 2:** Copper content in the green fodder of field pea/oat mixtures (means across 2010–2012), mg kg^−1^ d.m.

Component share in the mixture, %	Harvest date		Means
	Field pea flowering stage	Field pea flat green pod stage	
Field pea in pure stand 100%	5.072	4.248	4.660
Oat in pure stand 100%	4.245	3.376	3.811
Field pea 75% + oat 25%	4.693	3.914	4.304
Field pea 50% + oat 50%	4.458	3.708	4.083
Field pea 25% + oat 75%	4.207	3.537	3.872
Means	4.535	3.757	-
LSD_0.05_			
Component share in the mixture			0.212
Harvest			0.130
Interaction			0.272

Statistical analysis demonstrated a significant effect of the experimental factors and their interaction on zinc content in the green fodder of field pea/oat mixtures (Table 3). Zinc plays a major role in plant metabolism. Plant growth and development are hindered by both zinc shortage and excess (Ociepa-Kubicka and Ociepa 2012). Although zinc is necessary in plant nutrition, plants growing in a polluted environment may accumulate high amounts of this element, which can pose a serious threat to the health of people and animals (Yu-Wei et al. 2013; Sharma et al. 2004; Srinivas et al. 2002). In the present study, the highest zinc content was recorded in field pea grown in pure stand, it being the lowest in oat. Also Adefarati et al. (2017) recorded a similar zinc content in green peas. However, the values are lower than standards set by WHO/FAO and are not harmful to humans or animals. According to Fytianos et al. (2001), Demirezen and Aksoy (2006), and Ali and Al-Qahtani (2012), zinc content in cereals is lower compared with beans and peas. In the experiment reported here, field pea mixed with oat increased zinc content in the green fodder of mixtures, the values being lower than in field pea but higher than in oat. This finding corresponds to values reported by Goliński et al. (2007) and Szpunar-Krok et al. (2009). Harvest date had a significant effect on zinc content in the green fodder of field pea/oat mixtures. A higher concentration of this element was recorded in mixtures harvested at the stage of field pea flat green pod compared with the flowering stage. A delay in harvest was followed by an increase in the plant content of zinc. As zinc is a microelement, its higher amount in green fodder is of great importance for human and animal health (Płaza et al. 2018; Adefarati et al. 2017; Chandra Sekhar et al. 2002; Zarcinas et al. 2004). In the present study, an interaction between the experimental factors was confirmed. The highest zinc content was recorded in the green fodder of field pea grown in pure stand and harvested at the stage of flat green pot, it being the lowest in oat grown in pure stand and harvested at the stage of field pea flowering. Of the examined mixtures, the highest concentration of zinc was determined in field pea/oat mixtures whose component shares were 75 + 25% and 50 + 50%, and which were harvested at the stage of field pea flat green pot.

**Table 3:** Zinc content in the green fodder of field pea/oat mixtures (means across 2010–2012), mg kg^−1^ d.m.

Component share in the mixture, %	Harvest date		Means
	Field pea flowering stage	Field pea flat green pod stage	
Field pea in pure stand 100%	41.08	56.37	48.73
Oat in pure stand 100%	29.08	38.23	33.66
Field pea 75% + oat 25%	39.23	50.32	44.78
Field pea 50% + oat 50%	36.97	48.78	42.88
Field pea 25% + oat 75%	33.12	40.54	36.83
Means	35.90	46.85	-
LSD_0.05_			
Component share in the mixture			2.31
Harvest date			1.43
Interaction			3.12

Lead and cadmium contents were insignificantly affected by the experimental factors and their concentrations in the green fodder of field pea/oat mixtures were too low to be determined by means of the emission spectrometer Perkin Elmer Optima 8300. Thus, they did not exceed the standards set for green fodder in the Official Journal of the European Union (2013). It was due to the fact that fields where the research was conducted were well away from roads and the green matter of field pea/oat mixtures was a safe cattle feed. The air and soil are major sources of heavy metals for plants (Adefarati et al. 2017; Brigide et al. 2014). Despite low Cd and Pb contents in the green fodder of field pea/oat mixtures determined in the present study, it is necessary to continually monitor and check plant-derived feeds in terms of their heavy metal content.

Statistical analysis confirmed a significant effect of the experimental factors and their interaction on chromium content in the green fodder of field pea/oat mixtures (Table 4). The lowest chromium content was recorded in oat cultivated in pure stand, it being the lowest in field pea cultivated in pure stand. Field pea mixed with oat significantly reduced chromium content in green fodder. Some studies demonstrated excessive Cr amounts in cereals and rice (Akinyele and Shokunbi 2015; Pirsaheb et al. 2016; Brigide et al. 2014; Ali and Al-Qahtani 2012; Abdulmojeed and Abdulrahman 2011). In contrast, legumes are characterised by a lower concentration of chromium (Brigide et al. 2014; Akinyele and Shokunbi 2015). In the current study, there was confirmed a significant effect of harvest date on chromium content in the green fodder of field pea/oat mixtures. A higher concentration of this element was recorded in the field pea/oat mixture harvested at the stage of field pea flat green pod versus the flowering stage, which can be explained by the fact that plants harvested at later dates take up more chromium from the soil (Adefarati et al. 2017). However, in the experiment discussed here, chromium content in the green fodder of field pea/oat mixtures harvested at the stage of flat green pod was not high and posed no threat to animals. Similar findings were reported by Adefarati et al. (2017), Ladipo and Doherty (2011), and Sobucola et al. (2010) who demonstrated that chromium content in the plants they tested (cereals and legumes) did not exceed WHO/FAO standards. Chromium levels in plants are affected by their maturity at harvest. Small amounts of chromium are essential for life as the element plays a significant role in the metabolic transformations of glucose, some proteins, and fats (Ociepa-Kubicka and Ociepa 2012; Sobucola et al. 2010). In the study presented here, an interaction between the experimental factors was confirmed. The highest chromium content was recorded in the green fodder of field pea grown in pure stand and harvested at the flowering stage or the flat green pod stage, and in field pea/oat mixtures whose component shares were as follows: 75 + 25% and 50 + 50%, and which were harvested at the stage of field pea flowering. The highest chromium content was determined in oat grown in pure stand and harvested at the stage of field pea flat green pod.

**Table 4:** Chromium content in the green fodder of field pea/oat mixtures (means across 2010–2012), mg kg^−1^ d.m.

Component share in the mixture, %	Harvest date		Means
	Field pea flowering stage	Field pea flat green pod stage	
Field pea in pure stand 100%	1.353	1.696	1.525
Oat in pure stand 100%	3.560	6.914	5.237
Field pea 75% + oat 25%	1.874	3.423	2.649
Field pea 50% + oat 50%	2.157	4.072	3.115
Field pea 25% + oat 75%	2.981	5.340	4.161
Means	2.385	4.289	-
LSD_0.05_			
Component share in the mixture			0.623
Harvest date			0.249
Interaction			0.872

Nickel content in the green fodder of field pea mixed with oat was significantly affected by the experimental factors and their interaction (Table 5). The lowest nickel content was recorded in field pea grown in pure stand, it being the highest in oat cultivated in pure stand. Ni levels determined in the present study did not exceed WHO/FAO standards (Adefarati et al. 2017). Small amounts of nickel are necessary for plant growth and development (Akinyele and Shokunbi 2015), the element being toxic when present in excessive amounts (Cabrera et al. 2003). It should be stressed that, in the study reported here, field pea mixed with oat contributed to a decline in the green fodder content of nickel. Harvest date significantly affected Ni content in the green fodder of field pea/oat mixtures. Nickel content was lower in plants harvested at the stage of field pea flowering compared with the stage of flat green pod. Also, Ladipo and Doherty (2011) as well as Sobucola et al. (2010) reported that nickel content in plants was influenced by their maturity stage at harvest. In the present study, interaction between the experimental factors was confirmed. The lowest nickel content was determined in the green fodder of pure stand field pea harvested at the flowering stage and the stage of flat green pod, and in field pea/oat mixtures with the following shares of components: 75 + 25% and 50 + 50% harvested at the stage of field pea flowering. Nickel content was the highest in oat harvested at the stage of field pea flat green pod.

**Table 5:** Nickel content in the green fodder of field pea/oat mixtures (means across 2010–2012), mg kg^−1^ d.m.

Component share in the mixture, %	Harvest date		Means
	Field pea flowering stage	Field pea flat green pod stage	
Field pea in pure stand 100%	1.557	1.972	1.765
Oat in pure stand 100%	3.282	5.705	4.494
Field pea 75% + oat 25%	1.926	2.747	2.337
Field pea 50% + oat 50%	2.215	3.538	2.877
Field pea 25% + oat 75%	2.743	4.216	3.480
Means	2.345	3.636	-
LSD_0.05_			
Component share in the mixture			0.593
Harvest date			0.189
Interaction			0.732

## Conclusions

Field pea grown in pure stand had the highest copper and zinc contents, and the lowest chromium and nickel contents. Field pea mixed with oat contributed to a significant decline in the green fodder content of heavy metals.

Field pea/oat mixtures harvested at the stage of field pea flowering contained more copper but less zinc, chromium, and nickel compared with mixtures harvested at the stage of field pea flat green pod.

Cadmium and lead contents in the green fodder of field pea mixed with oat were insignificantly affected by the experimental factors, and were too low to be determined by means of the emission spectrometer Perkin Elmer Optima 8300.

Green fodder of field pea mixed with oat, whether harvested at the flowering stage or the stage of field pea flat green pod, was safe for cattle because it did not contain excessive amounts of heavy metals.

Despite a low heavy metal content in the green fodder of field pea mixed with oat, continual monitoring of these elements is recommended.
